# Impact of the Patient–Doctor Relationship on Treatment Outcomes in Children with Type 1 Diabetes: A Meta-Analysis and Systematic Review

**DOI:** 10.3390/children11091041

**Published:** 2024-08-26

**Authors:** Cristina Stefanescu, Denisa Boroi, Claudia Iuliana Iacob, Victorița Stefanescu, Aurel Nechita

**Affiliations:** 1 Faculty of Medicine and Pharmacy, “Dunărea de Jos” University, 800010 Galați, Romania; cristina.stefanescu@ugal.ro (C.S.); victorita.stefanescu@ugal.ro (V.S.); aurel.nechita@ugal.ro (A.N.); 2 Faculty of Psychology and Educational Sciences, University of Bucharest, 050663 Bucharest, Romania; denisa.boroi@yahoo.com; 3 Laboratory of Health Psychology and Clinical Neuropsychology, Department of Applied Psychology and Psychotherapy, Faculty of Psychology and Educational Sciences, University of Bucharest, 050663 Bucharest, Romania

**Keywords:** type 1 diabetes, pediatric diabetes, patient–doctor relationship, adolescence, children, meta-analysis

## Abstract

(1) Background: Despite the recognized importance of the patient–doctor relationship (PDR) for pediatric diabetes management, the literature presents diverse and emerging findings regarding its impact on treatment outcomes for children with type 1 diabetes (T1D). (2) Methods: Using a meta-analytic approach, a comprehensive search for relevant studies was conducted across major databases, from the earliest study to June 2024. Inclusion criteria were studies on PDR and T1D outcomes in underaged individuals, providing quantitative results. (3) Results: Fifteen reports were included, showing a small but significant overall effect size (r = 0.165, *p* < 0.05) of PDR on T1D outcomes. Moderator analyses revealed significant associations from elements of PDR, duration of diagnosis, outcome assessment methods, information reporters, and being Caucasian. Demographic variables like gender, age, not being Caucasian, and caregiver’s marital status did not significantly impact the association. Specific elements of PDR, such as clinician’s objectives, communication, partnership, respect, and supportive care, showed significant positive effect sizes, while telecommunication did not. (4) Conclusions: A strong PDR is essential for managing diabetes in children, particularly in the early years of diagnosis. Future studies should use quantitative designs and include diverse demographics to better understand PDR’s connection to T1D outcomes.

## 1. Introduction

Type 1 diabetes (T1D) is a chronic condition that significantly impacts the lives of children and their families [1]. Managing T1D requires rigorous adherence to treatment protocols, including regular blood glucose monitoring, insulin administration, and lifestyle adjustments [1]. The patient–doctor relationship (PDR) has been recognized as a crucial factor in influencing treatment adherence and outcomes in chronic conditions, including T1D [2]. The PDR refers to the interactions and bonds formed between a patient and their healthcare provider, encompassing communication, trust, and mutual respect, and leading to improved patient outcomes [3,4]. The theoretical underpinning of this study is based on the biopsychosocial model, which posits that health outcomes are influenced by the complex interplay of biological, psychological, and social factors [5]. In the context of T1D, the PDR serves as a critical social factor that can impact psychological well-being and, consequently, biological health outcomes through improved adherence to treatment and effective disease management.

Previous research highlights the importance of a strong PDR in improving health outcomes for patients with chronic illnesses. Most studies were performed on adults with diabetes, showing that glycemic control, treatment adherence, and psychological well-being are impacted by a solid PDR [6,7,8]. Research on children with diabetes and PDR is less advanced. However, there is evidence that children who perceive their doctors as supportive and communicative are more likely to adhere to their treatment plans, resulting in improved HbA1c levels [9]. Ref. [10] found that involving children in discussions about their treatment and actively engaging them in their care improved their overall glycemic control. Positive interactions between patients and their healthcare providers significantly improve adherence to treatment among adolescents with T1D. This adherence is needed for maintaining optimal blood glucose levels and preventing complications [11].

The relationship between PDR and T1D outcomes has been analyzed by three reviews. The first systematic review [12] demonstrated that effective provider–patient interactions positively influence self-care and clinical outcomes in diabetes care. Moreover, ref. [13] emphasized the potential of enhancing PDR to improve adherence among adolescents with T1D, underscoring the role of healthcare providers in fostering supportive relationships that encourage treatment adherence. The last review (to our knowledge) was carried out in 2018 [14] and it highlighted that improved communication between adolescents with T1D and their providers led to better management of the disease and a reduction in the incidence of complications. The results of these reviews are also supported by recent data, validating the effect of PDR and proving its consistency [9,15].

Despite the recognized importance of PDR, the literature presents divergent findings regarding its impact on treatment outcomes for children with T1D. Some studies report significant positive effects, while others find minimal or no association. For example, while some research indicates that a good PDR can lead to better glycemic control and diabetes management [9], other studies suggest that these relationships might be influenced by various moderating factors such as age, gender, and socio-economic status [4,14].

Given the diverse and emerging findings in the literature and the existing knowledge gap regarding the way PDR impacts treatment outcomes in children with T1D, this study employs a meta-analytic approach to (a) determine the overall effect size of the patient–doctor relationship on treatment outcomes; (b) quantify the heterogeneity of the studies; and (c) estimate the publication bias within this field. This approach provides a more robust, quantitative view of the phenomenon and includes a broader range of studies, incorporating more recent research.

## 2. Materials and Methods

### 2.1. Identification and Study Selection

**Figure 1:**
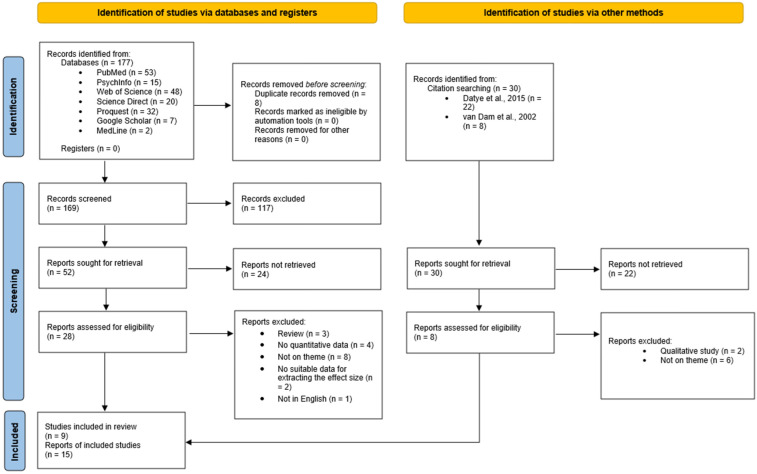
PRISMA flow chart: identification and study selection. Note: the referenced studies can be found at [12,13] in the reference list.

Potentially relevant studies were searched across major databases (PubMed, Science Direct, PsychInfo, Web of Science, Proquest, Google Scholar, MedLine) from June 3 to June 29, 2024 (see Figure 1 for more details regarding each database search). Additional studies were extracted by DB from previously conducted reviews in this field [12,13], during the same period of time. The Preferred Reporting Items for Systematic Reviews and Meta-Analysis (PRISMA) [16] was used for reporting the research strategy of the studies. In order to select the relevant studies, after the screening procedure, two independent reviewers (DB and CS) analyzed each full-text study and discussed any discrepancies in the assessment. The whole procedure was supervised by the third reviewer (CI). The search string used was: (patient-doctor relationship OR patient-physician relationship OR patient-provider relationship) AND (type 1 diabetes OR acceptance of the diagnosis OR treatment adherence OR glycemic control) AND (children OR adolescent* OR teenagers OR youth). 

### 2.2. Study Inclusion and Exclusion Criteria 

For this study, the following inclusion criteria were used: (1) Studies focusing on the patient–doctor relationship and glycemic control/adherence to treatment/acceptance of diabetes/management of diabetes in children with type 1 diabetes, with a correlational, quasi-experimental, or experimental design. (2) Studies that assessed the patient–doctor relationship using a psychometric instrument. (3) Studies that provided quantitative results related to glycemic control, treatment adherence, diabetes acceptance, or diabetes management. (4) Studies that provided data to calculate effect size. (5) Studies conducted on children/individuals under 18 years old diagnosed with type 1 diabetes or on their family members or doctors. (6) Studies written in English.

Studies that investigated PDR in adult populations and qualitative studies were not included. Studies that did not provide the necessary quantitative data for analysis or were not published in English were excluded, recognizing this as a potential limitation.

### 2.3. Moderators

Various categorical and continuous (numerical) moderators were extracted for analysis, including the following:

- Elements of PDR, categorized into six groups: clinician’s objectives (1 study), communication (5 studies), partnership (2 studies), respect for the patient and doctor’s agreeableness (2 studies), patient satisfaction with the relationship (2 studies), and telecommunication (3 studies).
- Time since diagnosis, divided into two categories: less than 5 years (4 studies) and more than 5 years (10 studies).
- Method of relationship evaluation: report (either by parents or doctor) used in 7 studies, self-report (5 studies), and evaluation by an external observer (3 studies).
- Method of outcome evaluation: objective measures recorded during the study (11 studies) and self-reports from participants (4 studies).

As continuous moderators within this conceptual framework, the following were included: (1) age, (2) percentage of females, (3) percentage of caregivers in a partnership (meaning they were married or in a relationship), (4) percentage of single caregivers, and (5) percentage of Caucasians or (6) percentage of participants of a race other than Caucasian (considering that most studies included a significantly lower percentage of participants from other races or cultures; in this group were African-American, Latino, and Asian participants).

### 2.4. Data Analysis and Meta-Analytic Procedures

The correlation coefficient was chosen to assess the effect size derived from statistical analyses. The effect size was extracted using Comprehensive Meta-Analysis software (CMA version 3.3.070, Bio-stat, Englewood, NJ, USA), as it is one of the most user-friendly meta-analysis software packages and its facilities are well recognized, especially among health care researchers [17]. Moreover, it has been widely used among recent meta-analyses [18]. Data reporting the mean, standard deviation, and sample size were converted into correlation indices for inclusion in CMA. Given the potential influence of each study’s heterogeneity on the results [19], the effect size was extracted based on a random effects model. The I^2^ statistic, indicating the proportion of total variance explained by heterogeneity, was used to assess variability between studies [19]. To assess the publication bias, we used the visual examination of the funnel plot and the “Classic fail-safe N” procedure [20].

## 3. Results

### 3.1. Literature Search Results 

The search strategy yielded 207 results. After removing duplicates, 199 studies were included for the next phase. Screening of titles and abstracts led to the exclusion of 88 studies and the full-text evaluation of 29 studies. In this stage, two independent reviewers assessed the potentially relevant studies meeting the inclusion criteria. Finally, nine studies were included in the meta-analysis [21,22,23,24,25,26,27,28,29]. The entire process is depicted in Figure 1. 

### 3.2. Study Characteristics 

In total, 15 effect sizes were extracted from the studies. The sample sizes of the included studies ranged from 56 to 1500 participants, with a total sample size of 7013 participants. The average participant age was between 13 and 16 years and the female percentage was between 44% and 57%. Most participants had a T1D diagnosis for more than 5 years and were Caucasians. In general, the participants were American (in 10 studies), European (2 studies), and Australian (1 study) citizens. Other details about the included studies can be found in Table 1.

### 3.3. Overall Effect Size

**Figure 2:**
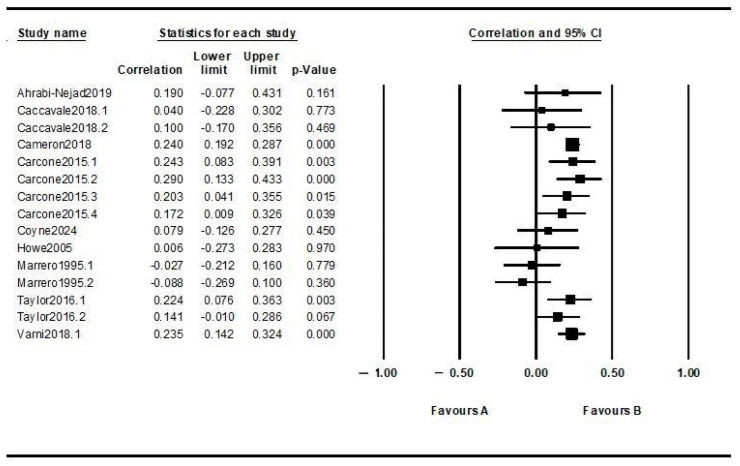
Forest plot for overall effect size. Note: study name = the included studies; statistics for each study = the statistical elements regarding the correlation quotient, the limits of the 95% confidence interval, and the level of significance (*p*); correlation and 95% CI = the graphic representation of the correlation quotients for each study within the 95% confidence interval; favours A = studies showing negative effects on the outcome; favours B = studies showing improvements in the outcome. The referenced studies can be found in the reference list at [21,22,23,24,25,26,27,28,29].

Meta-regression analysis based on the 15 extracted effect sizes showed a significant impact of the patient–doctor relationship on diabetes outcomes (including all types of results), with a correlation index of 0.165, *p* < 0.001, 95%-CI: [0.11, 0.21] (see Figure 2). The heterogeneity analysis indicated significant heterogeneity between the included studies (I^2^ = 45.388, *p* = 0.02), justifying the continuation of analyses.

Diabetes management was significantly associated with the patient–doctor relationship, with a correlation of 0.152, 95%-CI: [0.06, 0.23], *p* = 0.001, across six effect sizes. Glycemic control was also significantly associated, with a correlation of 0.152, 95%-CI: [0.06, 0.23], *p* < 0.001, across nine effect sizes, meaning a good patient–doctor relationship was connected with significant improvements in diabetes management and glycemic control (see Table 2). After removing outlier studies, the results remained significant with a slightly higher correlation index of 0.242, *p* < 0.001. 

### 3.4. Moderators Analysis

Separate analyses of the categorical moderators revealed relevant findings (see Table 3). Specific elements of the patient–doctor relationship had a significant impact, with *p* < 0.001 and an effect size of 0.175. The only non-significant element was telecommunication (*p* = 0.45). The time since diagnosis was significant (*p* = 0.001), with the most significant impact for diagnoses received within the last 5 years (correlation of 0.227, *p* < 0.001), while diagnoses older than 5 years had a lower effect (correlation of 0.145, *p* < 0.001). The method of relationship evaluation influenced the results (*p* < 0.001), with self-reporting by patients having the highest relevance (correlation of 0.219, *p* < 0.001), followed by reporting by the doctor/parent (correlation of 0.137, *p* = 0.001). External observer evaluations were not significant (*p* = 0.340). The method of outcome evaluation showed similar associations for both objective records and self-reports (correlations of 0.158 and 0.174, respectively, *p* < 0.01). When the participants in the study were the parents, the relationship between the study variables increased most significantly (r = 0.209, *p* < 0.001), compared to situations where the participants were either the patients (r = 0.114, *p* = 0.008) or the attending doctors (r = 0.199, *p* = 0.004), with the latter two instances yielding approximately equal results.

After analyzing the continuous moderators, gender distribution, reflected here in the percentage of girls, did not emerge as a significant moderator (*p* = 0.06). The same was true for the other moderators: the relationship status of the primary caregiver (*p* = 0.88 for both caregivers in a partnership and those who are single) and race (*p* = 0.182 for the percentage of Caucasians, *p* = 0.185 for other races). For details, see Table 2.

### 3.5. Publication Bias

**Figure 3:**
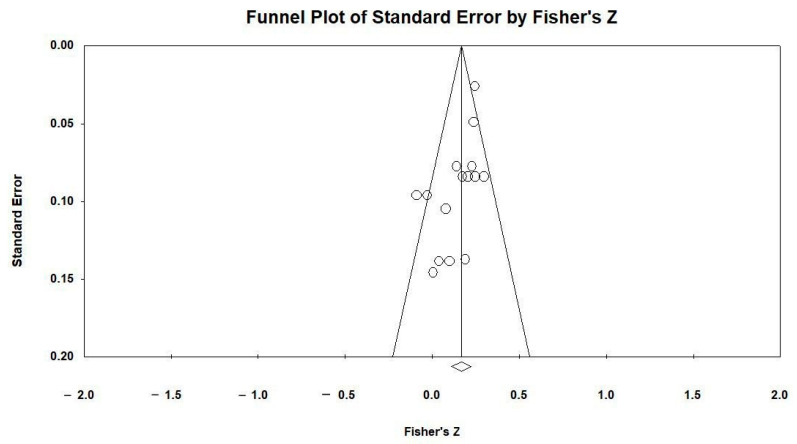
Funnel plot of Fisher’s z errors used to assess the potential for publication bias and the variability of effect sizes across studies. Note: Standard error = the precision of the effect size estimate from each study; Fisher’s z = the transformed correlation coefficients used to standardize the effect size.

Publication bias was assessed using two methods. Visual examination of the funnel plot indicated missing studies marked by black dots (Figure 3). The “Classic fail-safe N” results suggested no significant publication bias, as a very large number of unpublished studies (approximately 254) would be required to invalidate the current results. 

## 4. Discussion

The PDR is essential for successful treatment in cases of pediatric T1D, and numerous factors contribute to such favorable outcomes. From exhibiting a supportive and respectful attitude towards the patient to effective communication, setting goals, and cooperation, all these elements are important to monitor to create a good relationship with the patient and achieve greater treatment efficiency. To date, no meta-analysis was performed to investigate these claims in the emerging quantitative literature, so this study filled the above-mentioned gap.

Overall, it was confirmed that a solid PDR is associated with multiple T1D outcomes in underaged individuals. Systematic reviews and other recent studies [11,30] emphasize the importance of adopting a peer-to-peer attitude, especially in the case of adolescents, as well as communicating in terms that are as close as possible to the patient’s level of understanding about the disease and its effects. Considering the specifics of the age group, they recommend taking courses on communication with pediatric patients, showing that doctors who have taken such courses had a success rate 1.62% higher than those who have not. Our study supports such results and, at the same time, extends them regarding the nuance it offers to communication. Thus, regarding the mode of communication, the present study indicates the lack of a significant impact of communication through technology, as opposed to face-to-face communication, which has significant positive associations with PDR. Additionally, the importance of communication is backed up with recent data showing significant improvements in the communication process between doctor and patient lead to better treatment outcomes and a higher quality of life for patients [25,29].

One explanation for this could be the small number of studies included for each category, but also the fact that it is not the means of communication that is important, but the presence of communication itself (a fact suggested by the significance level of the category reflecting the presence of communication). Additionally, it is possible that in the case of communication through technological means, information related to treatment or elements necessary for creating a solid bond (such as physical presence or non-verbal cues) may be lost in the virtual environment, due to the larger volume of stimuli that the patient (or even the doctor) has to manage, so this form of communication does not produce improvements. The studies included in our meta-analysis regarding telecommunication come from a large time frame, from 1995 [27], to 2005 [26], and 2019 [21], and they all admit that physical contact may be a crucial factor in the pediatric diabetes treatment, particularly as they found that communication by virtual means had nearly insignificant effects. Careful consideration is needed, though, when interpreting this result, as although recent reviews regarding the use of telemedicine in pediatric chronic diseases [31,32] found no impact on the quality of life or treatment outcome, they showed telemedicine is associated with numerous advantages (e.g., fewer hospitalizations, fewer emergency cases, or better mental health). Thus, when deciding whether to implement telemedicine or not, the decision should be dependent on a certain objective. 

The PDR has a stronger association with treatment outcomes when diagnosis was more recent, with the first five years from diagnosis being particularly relevant. These initial years are vital for forming a bond with the doctor, accumulating information about the diagnosis, and dealing with potential complications until full acceptance of the diagnosis and treatment is achieved [33]. During this period, the need for information, support, and confidence in medical services is more pronounced, and the treating doctor and the relationship with them play a significant role in achieving favorable outcomes. Another explanation could be the adjustment and stabilization of symptoms in the period following the diagnosis, while over a longer period, following adherence to the treatment, the symptoms may decrease in intensity. Of note are also the results of [24], which can explain the mechanisms behind PDR, through another pathway, i.e., the one of caregivers. As such, their data, along with recent data [34], suggest the relationship the doctor forms with the caregiver is also crucial in encouraging the child to cooperate with the medical team, to help with important information or with managing the symptoms while the child is at home. Furthermore, this is more relevant as they included participants with a diagnosis under five years, suggesting this is also the period of creating a bond with the parents and assisting them in adapting to the diagnosis. Of all the included studies in our paper, the majority of studies reported the involvement of parents, with some even observing that the over-involvement of parents in treatment could negatively impact the engagement of children in the PDR [22,24,25,28], hence indicating the need for providing psychoeducation for parents. 

The way the relationship was assessed in studies was also significant, with self-reporting by patients being the most relevant, followed by reporting by the doctor or parent. Therefore, for the PDR to impact diabetes management, it is essential that the patient, first and foremost, perceives a good relationship with the doctor. The doctor is the primary actor involved in managing both the symptoms of diabetes and establishing a direct connection with the patient. This is supported by the lack of significance in evaluations by an uninvolved observer (although this could also be due the small number of studies that used this evaluation method).

In contrast to the assessment of the relationship, the method of evaluating diabetes showed similar results for both objective recordings and self-reports. This could be due to the objectivity of glycemic indicators, considering that glycemic values were the basis for self-reports as well. However, caution is needed before drawing a conclusion in this case, as only four studies relied on self-reporting [21,23,28,29], which could have influenced the results. 

Surprisingly, when the parent was the person providing the study results [24,28], the PDR had the greatest effect size on diabetes-related outcomes. In contrast, when the doctors or patients provided the necessary information for the study [23,26], the effect sizes were approximately equal. Consequently, two mechanisms might explain these results: (1) the parent might not always be aware of the disruptions that can occur in the PDR or might view the relationship more positively; or (2) the attitudes and perceptions of the parent toward the doctor or the child might influence the symptoms manifested by the child or the doctor’s involvement in the relationship. Additionally, the parent’s willingness to participate in the study may also indicate greater parental involvement in the child’s treatment, which could be an indicator of better care received by the child. However, this is only a tentative interpretation, and further studies comparing reporting differences within the same participant group are necessary to confirm or refute this explanation.

At the same time, the data showed that gender distribution was not relevant, meaning that the PDR is associated with diabetes-related outcomes equally for both girls and boys. Although some studies included here did find a better treatment response for girls [23,28], this was not supported at a meta-analytic level, which means the effect shown in the studies was rather due to the particularities of those samples. Additionally, the lack of significance of age, the marital status of the caregiver, or the race of the participants indicates that the same holds true for other demographic variables. The only exception here was being Caucasian, which proved significant. This could be due to the diversity of cases in Caucasian participants, as most studies were conducted on Caucasian people, whereas participants of other races or cultures were found in a disproportionately lower percentage. This could also mean that Caucasian children were more likely to be diagnosed with T1D, as they had more resources available for accessing the medical services. As such, this highlights the need to include more diverse participants in the studies, as well as the need to facilitate the access to medical services for all races and cultures.

Therefore, the way PDR is associated with diabetes-related outcomes seems to be consistent regardless of race, age, or the parent’s marital status in children, with the most important aspect being the direct involvement of the actors in the relationship (considering that only variables measuring such an aspect were significant). Another explanation could be the greater genetic influence in the case of diabetes [35], meaning that environmental influences might pertain more to other direct aspects related to the patient’s characteristics or the immediate environment, rather than more general variables that do not directly affect the participant and their adherence to treatment. In these cases, the literature shows very varied results, with studies indicating that socio-economic factors and access to medical care have a significantly greater impact on diabetes management than ethnicity itself [36]. Furthermore, regarding the marital status of the caregiver, while some studies have found an association between this and the outcome of diabetes treatment [36], others have not found such a connection, showing that the basis of a significant relationship might be parental support, active parental involvement, and the level of family stress [4]. Thus, the results of the present study support this hypothesis, and future studies could explore the mechanisms and differences involved in more detail. 

### 4.1. Limitations

The results highlight the limitations of the current state of knowledge regarding the impact of the PDR on the management of diabetes in children. It seems that few quantitative data have analyzed these aspects, as reflected in the relatively small number of studies included in the meta-analysis. This could have affected the results obtained here. While qualitative data provide valuable insights into the processes and manifestations occurring in these situations, they may have different effects at the group level. Therefore, more studies are needed to quantitatively measure the PDR using psychometric tools. This limitation also affects the generalizability of the results from the present meta-analysis, as the moderator analyses were sometimes based on small effect sizes.

Data about family environment, education level, socio-economic status, and the presence of social support—demographic elements that can influence treatment [37]— are missing from most studies examining the PDR and diabetes management. Therefore, no conclusions can be drawn regarding important variables that impact various health domains and the particularities of the PDR in managing pediatric diabetes. As a result, this lack of diverse demographic elements limits the generalizability of the results, as the impact of the PDR may vary significantly across different population groups. For instance, socio-economic status can affect access to healthcare resources, medication adherence, and the ability to follow treatment plans [37,38], thereby influencing the overall effectiveness of diabetes management. Similarly, educational level might determine a patient’s understanding of the disease [39] and the importance of maintaining a strong PDR. Moreover, family environment and social support play crucial roles in managing chronic conditions [24,39], particularly in pediatric populations, where parental involvement is needed in order to access medical services or the treatment recommendations. Without considering these variables, the findings of the meta-analysis may not accurately reflect the experiences of underrepresented groups, leading to potential biases and gaps in the application of the results to diverse patient populations. This means it is difficult to implement strategies that are effective across different demographic segments, ultimately impacting the quality of diabetes care and outcomes.

### 4.2. Future Research Directions

Future studies need to address these gaps in the literature by adopting a quantitative design and using established psychometric tools. This would strengthen the existing information and increase the generalizability of the results. Additionally, including demographic variables such as socio-economic status, family climate, and the presence of other diagnoses would be helpful for understanding how the relationship manifests in relation to these factors. These aspects could contribute to a deeper understanding of the topic and the adaptation of potential interventions. This is more significant as data show the cultural skills of the doctor are related to a better health outcome in patients of a different ethnic group [28]. In consequence, future studies could enhance sample diversity by adopting community-based recruitment, using a stratified sampling method, using targeted advertising, conducting research across varied locations, partnering with various educational and professional institutions, utilizing national databases in order to select the needed participants, offering flexible participation options, or providing socio-economic incentives. In doing so, future research could substantially enhance the chances of having more diverse and inclusive samples, thus offering clearer details regarding how the PDR and pediatric diabetes management manifests in the case of different socio-economic groups of patients. Furthermore, better guidelines for treatment in these cases could be developed, as the current ones offer scarce information related to this topic [34]. 

### 4.3. Conclusions

As the data showed, most variables moderating the associations between the PDR and diabetes outcomes are related to the attitudes/behaviors adopted by the doctor, specific disease aspects (e.g., duration), patient-specific aspects (how they perceive the doctor), or how these aspects were assessed. Demographic data proved insignificant in this case, necessitating future studies to clarify the contribution. The current data are the first to quantify the PDR and its connection with treatment adherence in children with type 1 diabetes through a meta-analytic design, providing clarification in this domain and future research directions. Integrating these findings into clinical interventions focused on building this relationship, as well as creating tools to measure it, are some ways in which these data could be applied. One way of doing so is, first and foremost, to create a safe and empathetic space for the patients so as they can form a feeling of trust in sharing personal information (without feeling shame or fear they will be judged) with the doctor. In other words, doctors should try to empathetically understand the patients’ difficulties, without the need to control the patients’ actions or the tendency to judge them, but to respect the patients’ difficulties and decisions, along with emphasizing the patients’ freedom of choice and personal autonomy. In turn, this could lead the patients to feel respected and cared for, and to engage more in the treatment plan, finally resulting in better health outcomes [4]. Additionally, special programs, such as centers or camps, can be developed where needed and recommended in order to achieve a better relationship and to emphasize other medical aspects that need to be addressed more carefully (e.g., finding a balanced diet, integrating physical activities in the daily routine). Their effectiveness regarding both diabetes outcomes and mental health of children (and their parents as well) are well supported by data [40,41,42], thus making them a good option to enhance the treatment results. 

In sum, the results presented in the current study provide valuable insights regarding the impact of the PDR on diabetes management in children with type 1 diabetes, while informing current healthcare practice and future pediatric diabetes strategies.

## Figures and Tables

**Table 1:** Study characteristics regarding the total number of participants, the percentage of girls, mean age, doctor–patient relationship element, type of outcome, outcome assessment, time since diagnosis, relationship assessment, type of rater, caregiver marital status, and the percentage of Caucasians or other racial groups.

Year	Author(s)	Title	*N*	% Girls	Mean Age	Relationship Element	Outcome	Outcome Assessment	Time since Receiving the Diagnosis	Relationship Assessment	Rater	Caregiver Marital Status	% Caucasian	% Other Racial Groups
2019	Ahrabi-Nejad	Provider-Patient Communication and Transition Readiness Among Adolescents with Type 1 Diabetes [21]	56	44%	15.5	communication	treatment adherence	self-report	>5 yrs	self-report	patient	73.7% partnered; 22.9% single	96.50%	5.40%
2018	Caccavale	Exploring the role of motivational interviewing in adolescent patient-provider communication about type 1 diabetes [22]	55	49.10%	14.8	communication	treatment adherence	self-report	>5 yrs	rater	patient	N/R	76.40%	23.60%
2018	Caccavale	Exploring the role of motivational interviewing in adolescent patient-provider communication about type 1 diabetes [22]	55	49.10%	14.8	communication	glycemic control	self-report	>5 yrs	rater	patient	N/R	76.40%	23.60%
2018	Cameron	The clinician factor: Personality characteristics of clinicians and their impact upon clinical outcomes in the management of children and adolescents with type 1 diabetes [23]	1500	50%	13	clinician target and agreeableness	glycemic control	record	>5 yrs	self-report	doctor	N/R	N/R	N/R
2015	Carcone	Multisystemic Therapy Improves the Patient-Provider Relationship in Families of Adolescents with Poorly Controlled Insulin Dependent Diabetes [24]	144	57%	14	partnership	glycemic control	record	<5 yrs	report	parent	59% single; 41% partnered	23%	77%
2015	Carcone	Multisystemic Therapy Improves the Patient-Provider Relationship in Families of Adolescents with Poorly Controlled Insulin Dependent Diabetes [24]	144	57%	14	respectful and supportive care	glycemic control	record	<5 yrs	report	parent	59% single; 41% partnered	23%	77%
2015	Carcone	Multisystemic Therapy Improves the Patient-Provider Relationship in Families of Adolescents with Poorly Controlled Insulin Dependent Diabetes [24]	144	57%	14	partnership	glycemic control	record	<5 yrs	report	parent	59% single; 41% partnered	23%	77%
2015	Carcone	Multisystemic Therapy Improves the Patient-Provider Relationship in Families of Adolescents with Poorly Controlled Insulin Dependent Diabetes [24]	144	57%	14	respectful and supportive care	glycemic control	record	<5 yrs	report	parent	59% single; 41% partnered	23%	77%
2024	Coyne	Intervention to Promote Adolescents’ Communication and Engagement in Diabetes [25]	94	53%	13	communication	glycemic control	record	>5 yrs	rater	patient	N/R	96%	4.20%
2005	Howe	Education and Telephone Case Management for Children With Type 1 Diabetes: A Randomized Controlled Trial [26]	75	45%	12.5	telecommunication	treatment adherence	record	<5 yrs	report	patient	N/R	55%	46%
1995	Marrero	Using Telecommunication Technology to Manage Children with Diabetes: The Computer-Linked Outpatient Clinic (CLOC) Study [27]	106	21%	13.3	telecommunication	glycemic control	record	>5 yrs	report	patient	N/R	96.22%	3.77%
1995	Marrero	Using Telecommunication Technology to Manage Children with Diabetes: The Computer-Linked Outpatient Clinic (CLOC) Study [27]	106	21%	13.3	telecommunication	glycemic control	record	>5 yrs	report	patient	N/R	N/R	N/R
2016	Taylor	Satisfaction with the Health Care Provider and Regimen Adherence in Minority Youth with Type 1 Diabetes [28]	169	52%	13.88	satisfaction with the provider	treatment adherence	record	>5 yrs	self-report	patient	67% married; 33% single	19%	82%
2016	Taylor	Satisfaction with the Health Care Provider and Regimen Adherence in Minority Youth with Type 1 Diabetes [28]	169	52%	13.88	satisfaction with the provider	treatment adherence	record	>5 yrs	self-report	parent	67% married; 33% single	19%	82%
2018	Varni	Diabetes Management Mediating Effects between Diabetes Symptoms and Health-Related Quality of Life in Adolescents and Young Adults with Type 1 Diabetes [29]	418	49.80%	16.3	communication	diabetes management	self-report	>5 yrs	self-report	patient	N/R	63.70%	36.30%

Notes: N/R = not reported; N/A = not applicable; yrs = years.

**Table 2:** The effect size obtained overall, for each outcome (diabetes management and glycemic control), and for continuous moderators (percentage of girls, mean age of participants, ethnicity, and marital status of the caregiver).

	β	SE	95%CI	I^2^	*p*	*p* Overall	Q
Overall effect size	0.165	0.004	[0.110; 0.218]	45.388	0.000 *	0.000 *	25.636
Effect size for each outcome							
Diabetes management	0.152	0.008	[0.064; 0.237]	0.000	0.606	0.001 *	2.719
Glycemic control	0.152	0.009	[0.069; 0.233]	62.144	0.007 **	0.000 *	21.133
Continuous Moderators							
%Girls	−0.209	0.110	[−0.426; 0.007]	0.000	0.058	0.056	23.263
Mean age	−0.264	0.379	[−1.007; 0.478]	0.004	0.485	0.399	14.695
Ethnicity							
%Caucasian	0.274	0.049	[0.177; 0.372]	0.001	0.000 *	0.182	17.377
% Other ethnicity	0.044	0.049	[−0.052; 0.142]	0.001	0.367	0.185	17.317
Marital status of caregiver							
%Single/Divorced	0.071	0.238	[−0.395; 0.538]	0.000	0.746	0.887	2.326
%Partnered	0.439	0.441	[−0.370; 1.358]	0.000	0.263	0.887	2.326

Note: β = slope; SE = standard error; 95%-CI = 95% confidence interval of the weighted mean effect size; I^2^ = the percentage of total variation across studies that is due to true differences in effect sizes; Q = variance across studies; *p* = level of significance for I^2^; *p* overall = level of significance for the overall effect. * *p* < 0.001; ** *p* < 0.01.

**Table 3:** Summary of studies for each subgroup of doctor–patient elements, time since diagnosis, type of outcome, PDR and outcome assessment, and type of rater.

		N	*R*	r 95%-CI	I^2^	*p*
Categorical Moderators						
Doctor–patient relationship elements	clinician target	1	0.240	[0.192; 0.287]	0.000	0.000 *
	communication	5	0.185	[0.111; 0.257]	0.000	0.000 *
	partnership	2	0.223	[0.111; 0.257]	0.000	0.000 *
	respectful and supportive care	2	0.232	[0.119; 0.339]	8.986	0.000 *
	satisfaction	2	0.183	[0.077; 0.284]	0.000	0.001 *
	telecommunication	3	−0.046	[−0.166; 0.074]	0.000	−0.452
Time since diagnosis	<5 years	4	0.227	[0.123; 0.327]	0.000	0.000 *
	>5 years	10	0.145	[0.077; 0.211]	59.224	0.000 *
Type of outcome	diabetes management	1	0.235	[0.054; 0.401]	0.000	0.012 **
	glycemic control	9	0.156	[0.080; 0.230]	62.144	0.000 *
	treatment adherence	5	0.141	[0.023; 0.255]	0.000	0.019 **
	report	7	0.137	[0.060; 0.213]	61.167	0.001 *
	self-report	5	0.219	[0.152; 0.284]	0.000	0.000 *
Outcome assessment	record	11	0.158	[0.092; 0.223]	58.794	0.000 *
	self-report	4	0.174	[0.049; 0.293]	0.000	0.007 **
Rater	doctor	2	0.198	[0.063; 0.326]	61.666	0.004 **
	parent	5	0.209	[0.115; 0.300]	0.000	0.000 *
	patient	8	0.114	[0.030; 0.196]	53.675	0.008 **

Note: N = number of studies including the outcome; r = the correlation quotient; r 95%-CI = 95% confidence interval of the weighted mean effect size; I^2^ = the percentage of total variation across studies that is due to true differences in effect sizes. * *p* < 0.001; ** *p* < 0.05.

## Data Availability

The data supporting this study are available upon request from the correspondent author.
